# PGRN Inhibits Early B‐cell Activation and IgE Production Through the IFITM3‐STAT1 Signaling Pathway in Asthma

**DOI:** 10.1002/advs.202403939

**Published:** 2024-10-16

**Authors:** Pingping Zhang, Changshun Ruan, Guangli Yang, Yaning Guan, Yin Zhu, Qian Li, Xin Dai, Yang An, Xiaoqi Shi, Pei Huang, Yan Chen, Zhixu He, Zuochen Du, Chaohong Liu

**Affiliations:** ^1^ Department of Pediatrics Affiliated Hospital of Zunyi Medical University Zunyi 563000 China; ^2^ Department of Pediatrics Guizhou Children's Hospital Zunyi 563000 China; ^3^ Collaborative Innovation Center for Tissue Injury Repair and Regenerative Medicine of Zunyi Medical University Zunyi 563099 China; ^4^ Chongqing Key Laboratory of Child Infection and Immunity Children's Hospital of Chongqing Medical University Chongqing 400014 China; ^5^ Zhanjiang Institute of Clinical Medicine Zhanjiang Central Hospital Guangdong Medical University Zhanjiang 524037 China; ^6^ Department of Hematology Central People's Hospital of Zhanjiang Zhanjiang 524037 China; ^7^ Department of Pathogen Biology School of Basic Medicine Tongji Medical College and State Key Laboratory for Diagnosis and Treatment of Severe Zoonotic Infectious Diseases Huazhong University of Science and Technology Wuhan Hubei China Hubei 430074 China

**Keywords:** asthma, BCR signaling, IFITM3, IgE, progranulin

## Abstract

Progranulin (PGRN) plays a critical role in bronchial asthma and the function of various immune cells. However, the mechanisms by which PGRN influences B‐cell receptor (BCR) signaling and immunoglobulin E(IgE) production are not fully understood. The study aimed to elucidate the molecular mechanisms through which PGRN affects BCR signaling, B‐cell differentiation, and IgE production. A PGRN knockout mouse model, along with techniques including flow cytometry, the creation of a bone marrow chimeric mouse model, total internal reflection fluorescence (TIRF), and Western blot (WB) analysis is employed, to investigate the link between PGRN and various aspects of B‐cell biology. It is discovered that the absence of PGRN in mice alters peripheral B‐cell subpopulations, promotes IgE class switching in a cell‐intrinsic manner, and affects B‐cell subpopulations. Additionally, PGRN modulates B‐cell functions by regulating BCR signaling pathways, metabolic processes, and the actin cytoskeleton during early B‐cell activation. Significantly, PGRN deficiency results in diminished production of NP‐specific antibodies. Moreover, it is found that PGRN inhibits B‐cell activation and IgE production through the PGRN‐IFITM3‐STAT1 signaling pathway. The findings provide new strategies for the targeted treatment of bronchial asthma, highlighting the crucial role of PGRN in B‐cell signaling and IgE production.

## Graphical abstract

Progranulin (PGRN) influences B‐cell receptor (BCR) signaling and immunoglobulin E (IgE) production, crucial in bronchial asthma. This study reveals that PGRN deficiency alters B‐cell subpopulations, enhances IgE class switching, and modulates BCR signaling via the PGRN‐IFITM3‐STAT1 pathway. These findings suggest potential therapeutic targets for asthma treatment.

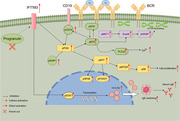

## 1 Introduction

Asthma is a common respiratory condition that affects children worldwide.^[^ ^1^ ^]^ Its primary clinical symptoms are increased airway inflammation and responsiveness, with several immune cells being pivotal in asthma progression.^[^ ^2^ ^]^ Notably, B cells play a key role in contributing to the development of asthma through the production of specific antibodies, particularly immunoglobulin E (IgE). The initial stimulation of B cells via the B‐cell receptor (BCR) signaling pathway triggers the B‐cell‐driven humoral immune response, which is closely associated with IgE production.^[^ ^3^ ^]^ Given the crucial role of IgE‐driven processes in both the onset and exacerbation of asthma, understanding the preliminary stages of BCR activation and their influence on IgE‐related asthma is essential.

The BCR, a transmembrane receptor expressed on the surface of B cells, consists of membrane‐bound immunoglobulins that recognize specific antigen epitopes.^[^ ^4^ ^]^ When antigens bind to the BCR, they induce structural alterations that lead to the recruitment and stimulation of internal signaling molecules such as Lyn, Syk, and Bruton's tyrosine kinase (Btk)^[^ ^5^ ^]^ These kinases act on essential signaling elements, initiating the activation of subsequent pathways, notably the phosphoinositide 3‐kinase (PI3K) and mitogen‐activated protein kinase (MAPK) cascade ^[^ ^6^ ^]^ Subsequently, B‐cell activation leads to several events, including clonal expansion, class‐switch recombination, and the differentiation of B‐cells into antibody‐secreting plasma cells or memory B cells.^[^ ^7^ ^]^ In the context of asthma, the early activation of B cells sets the stage for the production of antigen‐specific IgE.^[^ ^7^ ^]^ The interactions between allergens and activated B cells result in the differentiation of B cells into plasma cells, which produce and secrete IgE.^[^ ^7^ ^]^ IgE, in turn, binds to the high‐affinity IgE receptor (FcεRI) on the surface of mast cells and basophils, priming them for subsequent allergic responses. Understanding the intricacies of early BCR activation is pivotal for delineating the mechanisms underlying IgE‐mediated asthma. By deciphering the key molecular events that govern this process, novel therapeutic targets to intervene with and modulate B‐cell responses may be identified, potentially offering innovative approaches for managing and treating childhood asthma.^[^ ^8^ ^]^

IFITM3 (Interferon‐Induced Transmembrane Protein 3) plays a crucial role in the innate immune system, primarily known for its effectiveness in inhibiting viral entry and replication within host cells.^[^ ^9^ ^]^ It also plays an instrumental role in the early activation of B cells, particularly in the transduction of BCR signaling.^[^ ^10^ ^]^ IFITM3 serves as a scaffold for PIP3, amplifying the PI3K signal, which is paramount for B‐cell functionality. This event impacts the activation, proliferation, antigenic response, and cellular adhesion of B cells.^[^ ^11^ ^]^ Concurrently, STAT1 (Signal Transducer and Activator of Transcription 1) is an important downstream signaling molecule of IFITM3,^[^ ^12^ ^]^ it also plays a critical role in B cell activation and functional regulation.^[^^]^ The presence of *STAT1* ^–/–^ mice suggests a potential involvement in IgE regulation, as these mice exhibit altered immune responses.^[^ ^14^ ^]^ However, the direct impact of the IFITM3‐STAT1 pathway on IgE production and allergic responses remains unconfirmed.

Progranulin (PGRN), also known as granulin epithelin precursor or PC‐cell‐derived growth factor, is a multifunctional glycoprotein involved in various biological processes, including cell growth, survival, and inflammation.^[^ ^15^ ^]^ Its pleiotropic nature has garnered considerable attention in the context of inflammatory diseases. Research indicates that PGRN is pivotal in shaping immune reactions and orchestrating inflammatory processes, making PGRN a compelling focus in the study of the underlying causes of asthma.^[^ ^16^ ^]^ Beyond asthma, the role of PGRN has been explored in depth in various other inflammatory diseases, including rheumatoid arthritis, inflammatory bowel disease, and disorders related to neurodegeneration.^[^ ^17^ ^]^ In these contexts, PGRN has demonstrated both pro‐inflammatory and anti‐inflammatory activity, underscoring its multifaceted and context‐specific role in immune modulation. Regarding the specific role of PGRN in asthma, emerging research has begun to elucidate its potential involvement. Recent studies have indicated that PGRN expression levels are altered in the bronchial tissues of asthmatic individuals, suggesting its association with disease development and severity.^[^ ^18^ ^]^ Moreover, PGRN's interactions with various immune cells, including eosinophils and T cells, have been implicated in the modulation of airway inflammation and hyperresponsiveness in asthma.^[^ ^19^ ^]^ As B cells play a central role in IgE production, investigating the interplay between PGRN and BCR signaling may provide critical insights into the mechanisms governing IgE‐mediated asthma. Elucidating the potential mechanism by which PGRN regulates B‐cell responses via BCR signaling could lead to the discovery of novel therapeutic strategies aimed at mitigating IgE‐related allergic responses in childhood asthma.

To investigate the role of PGRN in asthma pathogenesis, we conducted experiments utilizing PGRN knockout (PGRN KO) mice. Our findings demonstrated that PGRN actively participates in the regulation of B‐cell differentiation, early B‐cell activation via BCR signal transduction, and cytoskeletal rearrangement, ultimately modulating B‐cell‐mediated immune responses. Notably, our research revealed an important mechanism through which PGRN influences asthma‐related processes. We found that PGRN can suppress the production of IgE, a crucial mediator of allergic responses, through the IFITM3/STAT1 signaling pathway. The suppression of IgE synthesis by PGRN indicates its potential as a novel therapeutic target for IgE‐driven asthma. By revealing the core molecular processes, we provide a deeper understanding of the pathogenesis of IgE‐driven asthma and suggest a promising direction for developing specific treatments. Our discoveries have substantial potential in paving the way for alternative therapeutic approaches for patients with IgE‐driven asthma, with the ultimate goal of reducing disease impact and improving the quality of life of these patients.

## 2 Results

### 2.1 PGRN Deficiency in Mice Alters Peripheral B‐Cell Subpopulations and Enhances IgE Class Switching

**Figure 1:**
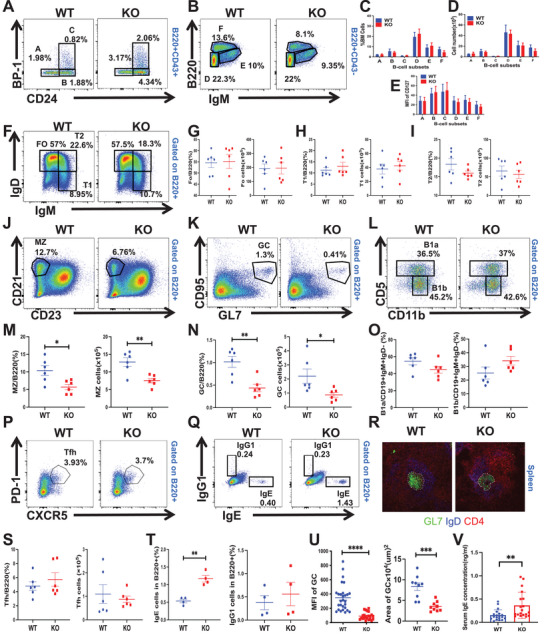
PGRN Deficiency in Mice Alters Peripheral B‐Cell Subpopulations and Enhances IgE Class Switching A–D) Flow cytometric analysis of various B‐cell subpopulations in the bone marrow of WT and PGRN KO mice, including A) pre‐pro‐B, B) pro‐B, C) early pre‐B, D) late pre‐B, E) immature B, and F) recirculating B cells. Data are presented as mean proportions (± SEMs) and absolute numbers of these bone marrow subpopulations within each B‐cell group (*n =* 6). E) Measurement of CD127 mean fluorescence intensity (MFI) across the B‐cell subsets identified in subpopulations A–F (*n =* 6). F–O) Flow cytometric analysis was performed to determine the percentage and total cell counts of follicular (FO), transitional 1 (T1), transitional 2 (T2), marginal zone (MZ), germinal center (GC) B cells in peripheral splenocytes, and B1a and B1b in the peritoneal cavity of WT and PGRN KO mice (*n =* 6). P) Flow cytometric analysis of T follicular helper (Tfh) within the peripheral splenocyte population in WT and PGRN KO mice (*n =* 6). Q) Representative flow cytometry plots showing IgE and IgG_1_ levels in B cells after 5 days of in vitro stimulation with anti‐CD40 antibody and IL‐4. R) Immunofluorescence staining was conducted on splenic sections from WT and PGRN KO mice, highlighting GC B cells using CD4 (red), IgD (blue), and GL‐7 (green). The scale bar indicates 100 µm. S) Percentage and cell count of Tfh cells gated from P(*n =* 6). T) Percentage of IgE^+^ and IgG_1_^+^ cells gated from Q (*n =* 4). U) Quantification of GC area and MFI from the spleen of WT and PGRN KO mice. V) Serum IgE concentrations in WT and PGRN KO mice were measured using ELISA (*n =* 19).

B lymphocytes originate from pluripotent stem cells in the bone marrow and progress through a series of antigen‐independent stages, including pre‐pro‐B, pro‐B, early pre‐B, late pre‐B, immature B, and recirculating B cells, corresponding to subgroups A through F, respectively.^[^ ^23^ ^]^ To explore the role of PGRN in B‐cell differentiation and development within the bone marrow, we generated PGRN KO mice and conducted a comparative analysis of their bone marrow cells with those from WT mice using flow cytometry. Early‐stage subgroups A (pre‐pro‐B), B (pro‐B), and C (early pre‐B) were distinguished using anti‐BP‐1 and anti‐CD24 antibodies, while B220 and IgM markers were employed to identify cells in stage D (late pre‐B), E (immature B), and F (recirculating B) subgroups. The analysis revealed no significant differences in the percentages or absolute numbers of cells in subgroups A, B, C, D, E, and F within the bone marrow between PGRN KO and WT mice (**Figure** **1**A–D). Additionally, we assessed the expression of CD127, a key molecule in bone marrow B‐cell development, and found no significant differences in CD127 expression across subgroups A‐F in either WT or PGRN KO mice (Figure 1E). These findings suggest that the deletion of the PGRN gene does not have a noticeable impact on B‐cell development and differentiation in the bone marrow.

The development and differentiation of B cells in the bone marrow leads to the production of naive B cells that migrate to peripheral lymphoid organs. Upon encountering antigens, these cells activate, multiply, and evolve into plasma and memory B cells. To discern PGRN's influence on peripheral B‐cell differentiation within the spleen, we undertook flow cytometry of nucleated spleen cells from WT and PGRN KO mice. Our analysis indicated consistent proportions and counts of cells within the follicular (FO) B‐cell, transitional 1(T1), and transitional 2(T2) categories (Figure 1F–I). Yet, in non‐immunized scenarios, both percentages and counts of marginal zone (MZ) B cells and germinal center (GC) B cells were notably reduced in PGRN KO mice relative to WT mice (Figure 1J,K,M,N). This data implies PGRN's regulatory role in MZ and GC B cell differentiation, potentially influencing GC B‐cell formation in mice. Additionally, we analyzed B1 cell staining within the peritoneal cavity, revealing no marked disparities between B1a and B1b cells across both mouse types (Figure 1L,O). We also probed variations in splenic follicular helper T (Tfh) cells across the two groups, finding no significant differences in Tfh cell proportions or counts (Figure 1P,S). Further detection of CD4, CD8, and Treg cell proportion in the spleen; a decrease in the proportion of CD4 and CD8 cells but an increase in Treg cells was detected in KO mice (Figure S1A,B, Supporting Information). The CD4 cells secreted less of IFNγ, although no statistically significant differences were observed in the levels of IL‐4 and IL‐17(Figure S1C,D, Supporting Information). Furthermore, we evaluated B cell capacity for IgE class switching in both groups. After stimulating B cells in vitro with IL‐4 and an anti‐CD40 antibody over 5 days, we determined the frequency of IgE^+^B220^+^ cells (Figure 1Q,T). Immunofluorescence of the spleen highlighted a decreased in GCs in PGRN KO mice compared to WT mice (Figure 1R,U). Serum IgE levels in both mouse types were also assessed, revealing heightened IgE levels in PGRN KO mice (Figure 1V), suggesting a superior IgE switching capability in these mice.

To confirm that PGRN intrinsically regulates B cells, we established a mouse bone marrow chimera model (Figure S1E, Supporting Information). Analysis revealed consistent FO B, T1 B, and T2 B cells between WT and PGRN KO mice within the CD45.2‐positive cell cohort of the recipient mice. Yet the percentages of GC B cells and MZ B cells within the CD45.2‐positive cell group were decreased in PGRN KO mice compared to WT mice (Figure S1F–M, Supporting Information). This underscores PGRN's influence on the differentiation of GC B cells and MZ B cells in the spleen is B cell‐intrinsically.

Taken together, our data indicates that PGRN plays an important role in maintaining the stability of peripheral splenic B cells in mice. PGRN intrinsically promotes the differentiation of MZ and GCB cells. PGRN deficiency in mice is associated with elevated serum IgE levels and an enhanced propensity for B cells to engage in IgE class switching.

### 2.2 PGRN Inhibition Enhances Early B‐Cell Activation by Modulating BCR Signaling

**Figure 2:**
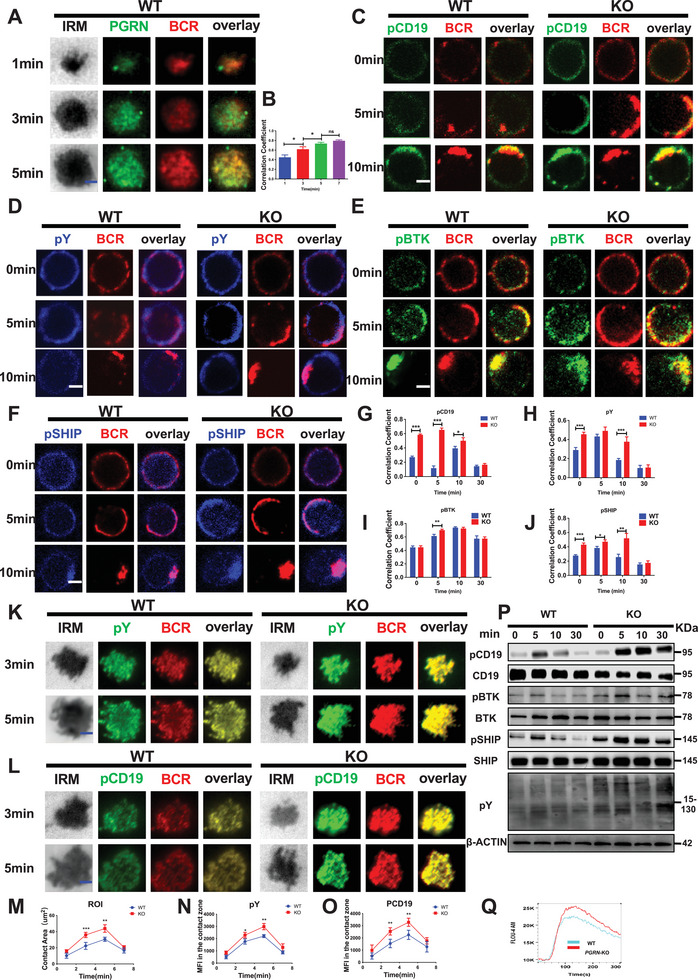
PGRN Inhibition Enhances Early B‐Cell Activation by Modulating BCR Signaling A,B) TIRF microscopy (TIRFm) was employed to visualize alterations in PGRN and BCR levels in WT murine B cells after in vitro stimulation with membrane antigen. The Pearson correlation coefficients between BCR and PGRN expression levels are presented (B). The scale bar indicates 2.5 µm. C–J) Colocalization of BCR with pCD19, pY, pBTK, and pSHIP in splenic B cells from WT and PGRN KO mice after in vitro stimulation with soluble antigens was analyzed. Colocalization coefficients were calculated using NIS‐Elements AR software. The scale bar represents 2.5 µm. K–O) TIRFm analysis was conducted to assess the levels of BCR, pY, and pCD19 during spontaneous fluctuations in splenic B cells from WT and PGRN KO mice following in vitro stimulation with membrane antigen. Changes in the contact area and mean fluorescence intensities (MFIs) of BCR, pY, and pCD19 were quantified using TIRFm. The scale bar indicates 2.5 µm. P) Western blot analysis was performed to measure the levels of pBTK, BTK, pCD19, CD19, pSHIP‐1, SHIP‐1, and pY in B cells from WT and PGRN KO mice after in vitro stimulation with soluble antigen, using β‐ACTIN or total protein as loading controls. Representative blots from three independent experiments are shown. Q) Representative calcium flux flow cytometry plots from three independent experiments demonstrate the effects of in vitro treatment with F(ab')_2_ Ig (M + G) on purified splenic B cells.

BCR signaling is essential for B‐cell proliferation, differentiation, survival, and activation.^[^ ^24^ ^]^ In this study, we investigated the impact of PGRN on BCR signaling in mice. We first examined the colocalization of PGRN with the BCR in activated B cells from WT mice and found significant colocalization at various time points, indicating the involvement of PGRN in early B‐cell activation (**Figure** **2**A,B). To further explore the role of PGRN in early B‐cell activation, we used confocal microscopy to observe changes in pCD19 and pY levels during early BCR activation in both WT and PGRN KO mice. We analyzed the colocalization coefficients of pCD19 and pY in relation to BCR activation and found higher Pearson correlation coefficients in PGRN KO mice, suggesting that PGRN can inhibit early B‐cell activation (Figure 2C,D,G,H).

To assess the impact of PGRN on early B‐cell activation, we utilized confocal microscopy to examine the colocalization of pBTK with the BCR in activated B cells. Our results showed that the colocalization of pBTK with the BCR was significantly increased in PGRN KO mice compared to WT mice, with elevated levels of pBTK also observed (Figure 2E,I). Additionally, we explored the colocalization of the negative regulatory molecule pSHIP with the BCR and the protein level of pSHIP following BCR activation. Unexpectedly, we found that the colocalization of pSHIP with the BCR was also increased in PGRN KO mice relative to WT mice (Figure 2F,J). Furthermore, the protein level of pSHIP was higher in PGRN KO mice (Figure 2P).

We also verified the changes in pCD19 and pY levels in WT and PGRN KO mice using total internal reflection fluorescence microscopy (TIRFm). Our findings demonstrated that in PGRN KO mice, the levels of IRM, pCD19, and pY were increased in B cells after membrane antigen stimulation, further confirming the impact of PGRN on B‐cell signal transduction (Figure 2K–O). Furthermore, Western blot analysis showed that the pCD19, pBTK, and pY levels were higher in PGRN KO mice than in WT mice after early B‐cell activation (Figure 2P).

Additionally, upon BCR stimulation, B cells undergo a rapid and significant increase in intracellular calcium concentration, which is attributed to both the influx of extracellular calcium and the release of calcium from intracellular stores.^[^ ^25^ ^]^ To investigate the impact of PGRN on this process, we assessed the change in the intracellular calcium level in B cells upon BCR activation. Notably, PGRN KO mice exhibited an increased rate of calcium influx (Figure 2Q).

In summary, our findings suggest that PGRN is involved in the transduction of BCR signaling after early B‐cell activation. Our results indicate that PGRN knockout leads to enhanced proximal BCR signaling during early activation.

### 2.3 PGRN Modulates B‐Cell Functions by Regulating BCR Signaling Pathways and Metabolic Activities

**Figure 3:**
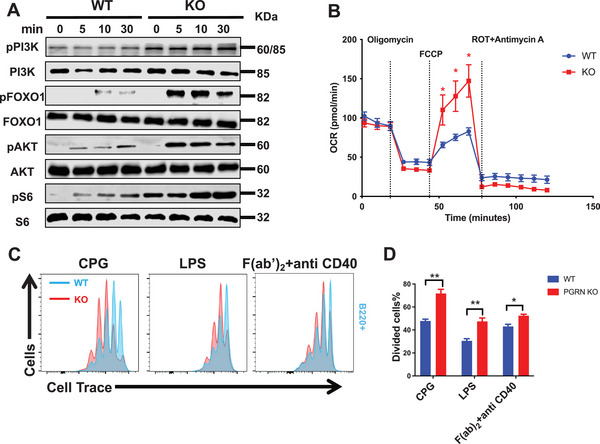
PGRN Modulates B‐Cell Functions by Regulating BCR Signaling Pathways and Metabolic Activities A) Western blot analysis depicting representative blots from three independent experiments, quantifying the expression levels of pPI3K, PI3K, pFOXO1, FOXO1, pAKT, AKT, pS6, and S6 in B cells from WT and PGRN KO mice after in vitro stimulation with soluble antigen. B) Oxygen consumption rate (OCR) was measured in purified splenic B cells from WT and PGRN KO mice following in vitro stimulation with either LPS or biotin‐conjugated F(ab')_2_ Ig(M+G), using a Seahorse XF24 Metabolic Flux Analyzer (*n =* 3). C,D) Flow cytometric profiles from three independent experiments are shown, illustrating the proliferation of purified B cells stained with CTV after stimulation with LPS, CpG, or F(ab')_2_‐Ig(M+G). Proliferation was quantified using flow cytometry analysis (*n =* 3).

After BCR activation, multiple downstream signaling pathways are activated. The activated BCR can activate various downstream signaling molecules, including Akt, mTOR, PKC, and NF‐κB, through PI3K‐mediated production of PIP3. These downstream signaling molecules play a crucial role in regulating B‐cell survival, proliferation, differentiation, and metabolism.^[^ ^26^ ^]^ To validate the impact of PGRN on B‐cell functions through its effect on BCR signaling, we conducted Western blot analysis to examine the expression of essential downstream signaling molecules in the PI3K signaling pathway, including pPI3K, pFOXO1, pAKT, and pS6. In PGRN KO mice, the protein levels of pPI3K, pFOXO1, pAKT, and pS6 were increased compared to those in WT mice upon stimulation with soluble antigens (sAg) (**Figure** **3**A). To investigate the effect of PGRN on B‐cell metabolism, we conducted a Seahorse assay upon stimulation with F(ab')_2_‐Ig(M + G) and found that PGRN KO B cells exhibited more robust ATP production and a higher maximal respiratory capacity than WT B cells (Figure 3B).

To investigate the effect of PGRN on B‐cell proliferation and apoptosis, we first assessed the proliferation and apoptosis of B cells under resting conditions. We initially established a bone marrow chimeric mouse model and, following BrdU injection examined the proliferation of CD45.2^+^ B cells and their subpopulations in WT and PGRN KO bone marrow chimeric mice. The results showed no significant difference in the proportion of BrdU^+^ cells between WT and PGRN KO mice (Figure S2A,D, Supporting Information). We then further analyzed the expression of Annexin V and Ki67 in various B‐cell subpopulations of WT and PGRN KO mice and similarly found no significant differences in the proliferation and apoptosis of B cells and their subpopulations between the two groups (Figure S2B,C,E,F, Supporting Information). Additionally, upon in vitro stimulation with LPS, CPG, or F(ab')_2_‐Ig(M+G), PGRN KO B cells exhibited a higher proliferation rate (Figure 3C,D).

Collectively, these results demonstrate that PGRN influences B‐cell functions by modulating BCR signaling pathways, leading to changes in downstream signaling molecules and altered metabolic activities, ultimately affecting B‐cell survival, proliferation, and differentiation. These results provide new insights into the role of PGRN in regulating B‐cell functions.

### 2.4 PGRN Modulates the Actin Cytoskeleton in B Cells following Early Activation by Inhibiting the MST1‐MTOR‐STAT1‐WASP Signaling Pathway

**Figure 4:**
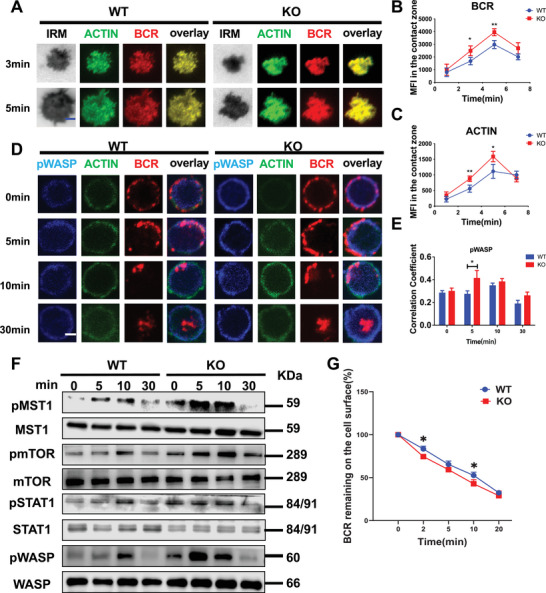
PGRN Modulates the Actin Cytoskeleton in B cells following Early Activation by Inhibiting the MST1‐MTOR‐STAT1‐WASP Signaling Pathway A–C) TIRF microscopy (TIRFm) was utilized to quantify alterations in IRM, BCR, and actin levels in splenic B cells from both WT and PGRN KO mice after in vitro stimulation with membrane antigen. The mean fluorescence intensity (MFI) of BCR (B) and actin (C) was determined using TIRFm. The scale bar represents 2.5 µm. D,E) Confocal microscopy was applied to examine the colocalization of BCR with pWASP in splenic B cells from WT and PGRN KO mice after in vitro stimulation with soluble antigens. Representative images of colocalized cells from each group are provided, with colocalization coefficients for BCR and pWASP calculated using NIS‐Elements AR software. The scale bar is 2.5 µm. F) Western blot analysis was carried out to measure the levels of pMST1, MST1, pmTOR, mTOR, pSTAT1, STAT1, pWASP, and WASP in B cells from WT and PGRN KO mice after in vitro stimulation with soluble antigen, with β‐actin or total protein used as controls. The blots shown are representative of three independent experiments. G) Flow cytometric analysis was performed to assess BCR internalization in splenic B cells from WT and PGRN KO mice following soluble antigen stimulation (*n =* 3).

Dynamic regulation of the actin cytoskeleton is crucial for BCR signal transduction and B‐cell function.^[^ ^27^ ^]^ To investigate actin cytoskeleton regulation, we stimulated WT and PGRN KO B cells with membrane‐associated antigens and used TIRFm to observe B‐cell spreading and BCR clustering. Initially, we evaluated the degree of F‐actin polymerization and BCR signaling by measuring the MFIs of F‐actin and the BCR in the contact area between WT and PGRN KO mouse B cells and membrane antigens. Our results indicated that the MFIs of F‐actin and the BCR in PGRN KO mice were increased at various time points compared to those in the WT group (**Figure** **4**A–C).

Considering that WASP is an important regulator of actin cytoskeleton dynamics, we investigated whether the disruption of F‐actin aggregation in PGRN KO mice is due to abnormal regulation of WASP. Our analysis revealed that the Pearson correlation coefficient between the BCR and pWASP was higher in PGRN KO mice than in WT mice, and Western blot analysis further confirmed an increase in WASP phosphorylation in PGRN KO mice (Figure 4D–F). Furthermore, we examined the levels of phosphorylated mTOR, MST1, and STAT1, which are involved in regulating the actin cytoskeleton via the MST1‐MTOR‐STAT1 signaling pathway.^[^ ^28^ ^]^ The levels of phosphorylated mTOR, MST1, and STAT1 were increased in PGRN KO mice compared to WT mice (Figure 4F). Furthermore, BCR internalization was similarly increased in PGRN KO mice (Figure 4G).

Overall, these findings demonstrate that PGRN modulates the actin cytoskeleton in B cells following early activation by inhibiting the MST1‐MTOR‐STAT1‐WASP signaling pathway.

### 2.5 PGRN Deficiency Leads to Impaired NP‐specific Antibody Production

NP‐KLH and NP‐Ficoll immunization is an approach to studying adaptive immune responses in mice.^[^ ^29^ ^]^ We sought to investigate the impact of PGRN on B‐cell function following NP‐KLH and NP‐Ficoll immunization in mice.

**Figure 5:**
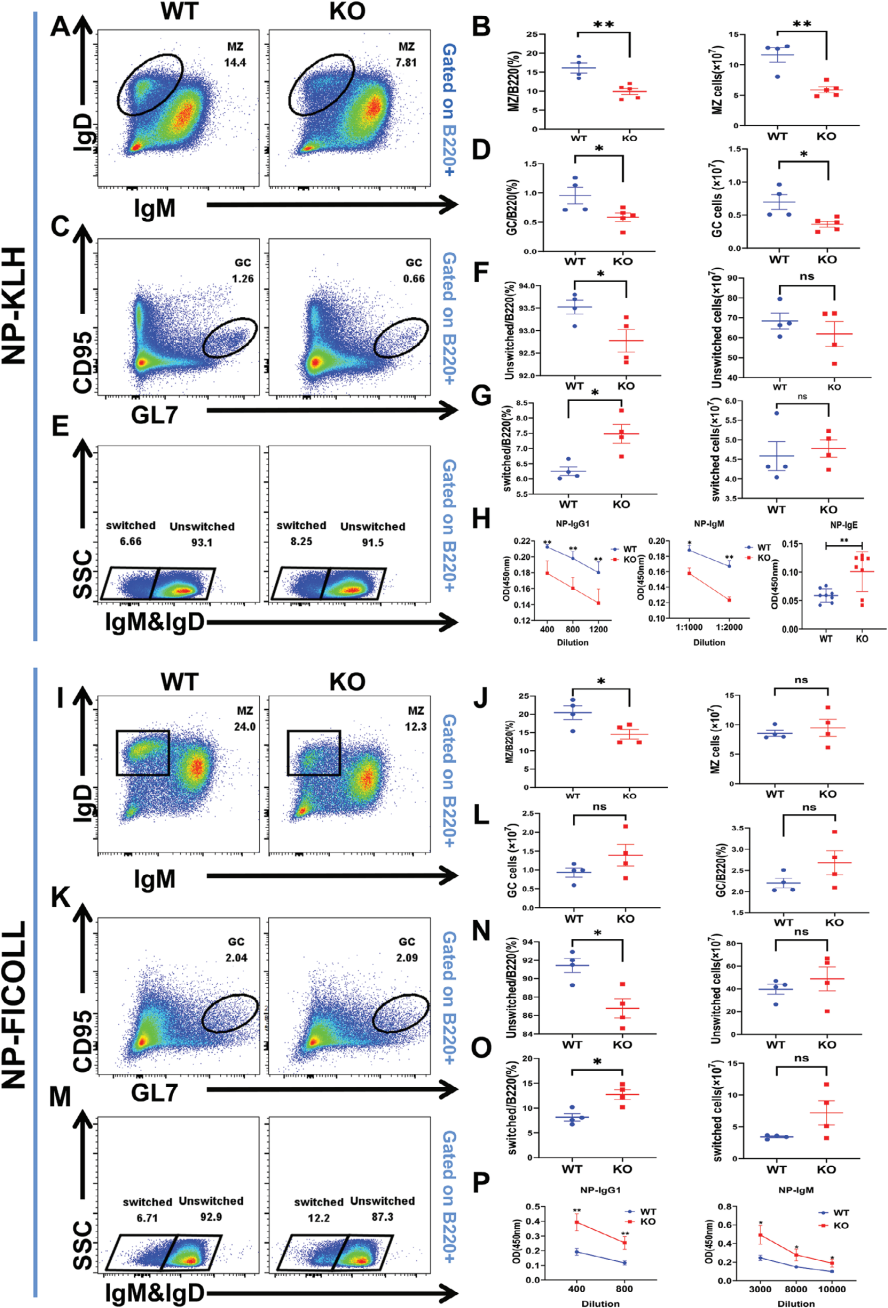
PGRN Deficiency Leads to Impaired NP‐Specific Antibody Production. A–G) Flow cytometric analysis was carried out on splenic cells isolated from NP‐KLH‐treated WT and PGRN KO mice. The identification of these cells was done using antibodies specific to the A) marginal zone, C) germinal center, and E) switched–unswitched B cells. Data are presented as mean proportions (± SEMs) and absolute numbers for these splenic cell subsets (B, D, F, G). H) ELISA was used to measure the optical density corresponding to NP‐specific IgG_1_, IgM, and IgE (dilution 1:50) serum antibody titers following secondary immunization (*n =* 8). I–O) Flow cytometric analysis was performed on splenic cells from NP‐Ficoll‐treated WT and PGRN KO mice, I) using antibodies specific to the marginal zone, K) germinal center, and M) switched/unswitched B cells. The data include the mean proportions (± SEMs) and absolute numbers of these splenic cell subsets (J, L, N, O) (*n =* 4). P) ELISA was used to quantify the optical density corresponding to NP‐specific IgG1 and IgM serum antibody titers (*n =* 5).

Prior to immunization, the percentages and absolute numbers of MZ and GC B cells in the spleens of PGRN KO mice were decreased compared with those in WT mice. However, upon NP‐KLH immunization, we did not observe substantial differences in the absolute numbers of T1, T2, and FO B cells even though the frequency of T1 B cells were decreased in KO mice (Figure S3A,D, Supporting Information), but we found significant reductions in the percentages and absolute numbers of two critical B‐cell subsets, MZ B cells (MZB) and GC B cells (GCB), in PGRN KO mice compared to WT mice (**Figure** **5**A–D). This finding indicates that PGRN deficiency impairs the differentiation or recruitment of MZ and GC B cells in the spleen following antigen exposure. Second, we evaluated the memory B cells and plasma cells in WT and PGRN KO mice after NP‐KLH immunization and found no difference between the populations of these B cells (Figure S3B,C,E,F, Supporting Information). Even though we found a decreased percentage of unswitched memory B cells and an elevated percentage of switched memory B cells, the absolute number of these two subpopulations were unchanged (Figure 5E–G). We also detected the production of NP‐specific IgM, IgG_1_, and IgE, which are essential components of humoral immunity. Notably, PGRN KO mice exhibited lower levels of NP‐IgG_1_ and NP‐IgM compared to the control group, while NP‐IgE levels were elevated, This suggests that PGRN knockout may lead to an imbalance in the humoral immune response to the NP antigen (Figure 5H).

Moreover, we immunized WT and PGRN KO mice with NP‐Ficoll to evaluate their T‐cell‐independent immune response capabilities. The results revealed no significant differences in cell numbers in various splenic B‐cell subpopulations, including MZ, FO, GC, T1, T2, memory, and plasma B cells, even though the percentage of MZ and unswitched memory B were decreased and switched memory B increased between WT and PGRN KO mice (Figure 5I–O; Figure S3G–L, Supporting Information). However, increased titers of NP‐specific IgM and IgG_1_ were observed in PGRN KO mice (Figure 5P), a finding inconsistent with the results in NP‐KLH immunized mice and thus elucidating the intricate function of PGRN in B‐cell immune responses.

Taken together, our study reveals that PGRN deficiency exerts varied effects on antibody responses in mice, depending on the type of antigen. Specifically, in PGRN KO mice, immunization with NP‐KLH resulted in reduced levels of NP‐specific IgM and IgG_1_ antibodies, along with an increase in IgE levels. This suggests that PGRN is essential for controlling T cell‐dependent antibody class switching and allergic responses. On the other hand, following NP‐Ficoll immunization, PGRN KO mice exhibited significantly higher levels of NP‐specific IgM and IgG_1_ antibodies, likely due to the diminished inhibitory influence of PGRN on B cell proliferation and activation. These findings underscore the complex regulatory roles of PGRN in immune responses triggered by different antigens, indicating that its function in immune modulation may vary based on the antigenic stimulus.

### 2.6 PGRN Inhibits B‐cell Activation and IgE Production Through the PGRN‐IFITM3‐STAT1 Signaling Pathway

**Figure 6:**
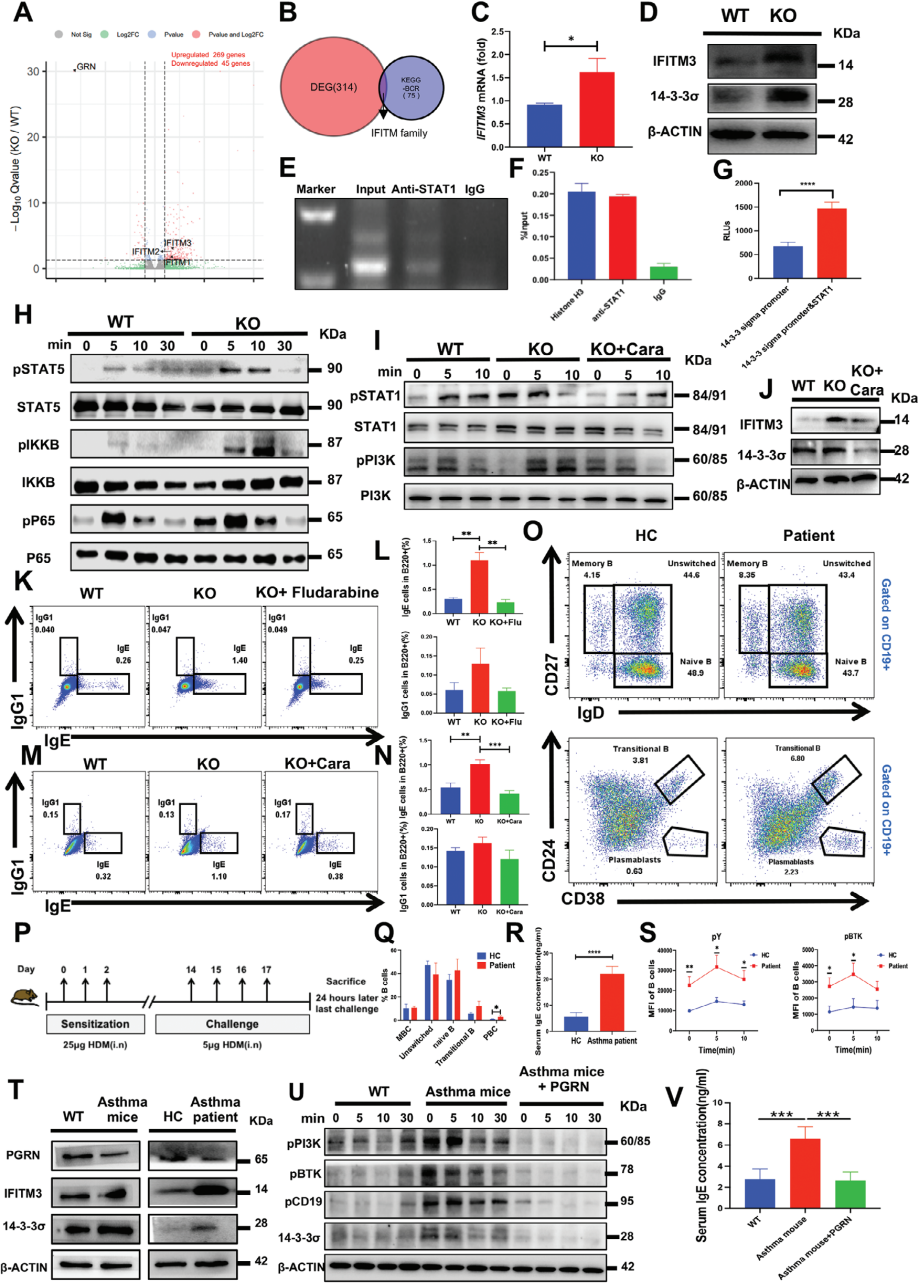
PGRN Inhibits B‐cell Activation and IgE Production through the PGRN‐IFITM3‐STAT1 Signaling Pathway. A) The volcano plot illustrates the differential gene expression between WT and PGRN KO mice, displaying log2 fold change (log2FC) on the *x*‐axis and –log10(Q) values on the *y*‐axis for each gene, with colors representing different levels of significance. B) Differentially expressed (DE) genes overlapping with BCR‐associated genes from the KEGG database are highlighted. C) The mRNA levels of *Ifitm3* in B cells from WT and PGRN KO mice are shown (*n* = 9). D) Western blot analysis shows the expression of 14‐3‐3σ and IFITM3 in B cells from WT and PGRN KO mice, using β‐actin as a loading control. The blots shown are representative of three independent experiments. E‐F) Chromatin immunoprecipitation (ChIP) was performed on purified splenic B cells from WT mice, followed by DNA purification and PCR (left) and real‐time PCR (right) using primers specific to the *14‐3‐3σ* promoter. G) A luciferase reporter assay was conducted in HEK293T cells to assess the regulation of *14‐3‐3σ* promoter activity, utilizing the pGL3‐14‐3‐3σ promoter and pAd5‐E1‐CMV‐STAT1 plasmid. H) Western blot analysis of pSTAT5, STAT5, pIKKβ, IKKβ, pP65, and P65 in B cells from WT and PGRN KO mice following in vitro stimulation with soluble antigen, with representative blots from three independent experiments. I) Western blot analysis of pSTAT1 and pPI3K levels in B cells from WT and PGRN KO mice treated with caraphenol A, using total protein as a control; representative blots are from three independent experiments. J) Western blot analysis showing the expression of 14‐3‐3σ and IFITM3 in B cells from WT and PGRN KO mice treated with caraphenol A, with β‐actin or total protein as controls; blots represent three independent experiments. K,L) Flow cytometry data showing levels of IgE and IgG_1_ in B cells from WT, PGRN KO, and PGRN KO mice treated with the STAT1 inhibitor Fludarabine after a 5‐day in vitro stimulation with anti‐CD40 antibody and IL‐4 (*n* = 3). M,N) Flow cytometry data displaying IgE and IgG_1_ levels in B cells from WT, PGRN KO, and PGRN KO mice treated with the IFITM3 inhibitor caraphenol A following a 5‐day in vitro stimulation with anti‐CD40 antibody and IL‐4 (*n* = 5). O) Flow cytometric analysis of B‐cell subsets within PBMCs from healthy controls (HCs) and asthma patients, quantifying the percentages of naive B, memory B, transitional B, and plasmablast cells (*n* = 6). P) A schematic overview of the house dust mite (HDM)‐induced asthma mouse model. Q) Proportions of B cells gated from O. R) Serum IgE levels in HCs and asthma patients (*n* = 10). S) PBMCs from HCs and asthma patients were labeled with APC‐anti‐CD19 antibody, followed by biotin‐conjugated F(ab’)_2_ Ig(M+G) and streptavidin, then stimulated at 37 °C. Following stabilization and permeabilization, mean fluorescence intensities (MFIs) of pY and pBTK were measured via flow cytometry (*n* = 10). T) Western blot analysis shows PGRN, 14‐3‐3σ, and IFITM3 expression in WT and asthma model mice, as well as in HCs and asthma patients, with β‐actin as a control; blots are representative of three independent experiments. U) Western blot analysis of B cells from WT, asthma model mice, and asthma model mice treated with PGRN in vitro, assessing pPI3K, pCD19, pBTK, and 14‐3‐3σ expression, using β‐actin as the control. Blots are from three independent experiments. V) ELISA analysis of serum IgE levels in WT mice, asthma model mice, and asthma model mice treated with PGRN in vivo (*n* = 6).

To delineate the underlying mechanisms by which PGRN inhibits B‐cell activation and IgE production, we first conducted mRNA sequencing of B cells derived from both PGRN KO and WT mice. Differential expression (DE) analysis revealed 314 genes with significant variations, with a false discovery rate (FDR) threshold of <0.05. Within this gene set, 269 genes exhibited elevated expression and 45 exhibited reduced expression in the PGRN KO group (**Figure** **6**A). A hierarchical clustering heatmap generated from the 314 differentially expressed genes identified two discrete clusters for the treated and control groups (Figure S4A, Supporting Information). Kyoto Encyclopedia of Genes and Genomes (KEGG) analysis highlighted notable modifications in the cytokine receptor pathway, hematopoietic cell lineage, and viral protein interaction with cytokine pathways (Figure S4B, Supporting Information). We intersected the DE genes with the BCR signaling pathway‐related genes in the KEGG database, determining that the IFITM family genes were the only genes impacted (Figure 6B). Previously, the literature reported that IFITM3 can amplify PI3K signaling in B cells by acting as a PIP3 scaffold^[^ ^30^ ^]^; thus, we further examined the mRNA and protein expression level of IFITM3 in PGRN KO B cells. The results showed that both mRNA and protein expression levels of IFITM3 were elevated in PGRN KO mice (Figure 6C,D), supporting the idea that PGRN may regulate B‐cell activation through IFITM3.

Previous literature has reported that IFITM3 overexpression can increase the STAT1 expression,^[^ ^31^ ^]^ and STAT1 is involved in the pathogenesis of SEA‐induced allergic rhinitis and IgE production,^[^ ^14^ ^]^ but the exact mechanism remains unclear. Our previous study demonstrated that the production of IgE is closely related to 14‐3‐3σ, which can promote IgE switching in B cells and the secretion of serum IgE.^[^ ^20^ ^]^ Then, we investigated the downstream effects of IFITM3 on B‐cell activation by assessing STAT1 phosphorylation. Intriguingly, we observed elevated protein levels of IFITM3 and 14‐3‐3σ in B cells of PGRN KO mice compared to those of WT control mice (Figure 6D). We further employed ChIP‐qPCR experiments to investigate whether STAT1 regulates the expression of 14‐3‐3σ by binding to its promoter. RT‐PCR showed PCR products in both the input and anti‐STAT1 ChIP samples, but not in the normal rabbit IgG ChIP sample (Figure 6E). Next, qPCR showed that a significant amount of DNA was immunoprecipitated in the anti‐STAT1 samples (Figure 6F). Additionally, we employed a luciferase reporter assay to further validate the regulation of 14‐3‐3σ transcription by STAT1. The results showed that STAT1 significantly upregulates the transcriptional activity of the *14‐3‐3σ* promoter in HEK293T cells (Figure 6G), indicating that STAT1 can bind to and enhance the transcription of the *14‐3‐3σ* promoter. We also measured the levels of various key proteins related to cytokine signaling pathways, such as STAT5, pSTAT5, pIKKB, IKKB, pP65, and P65, and found marked increases in these levels in PGRN KO mice (Figure 6H). After in vitro treatment with the IFITM3 inhibitor caraphenol A, the IFITM3, 14‐3‐3σ, pPI3K, and pSTAT1 were restored to normal levels (Figure 6I,J). This finding further substantiates the role of PGRN in regulating STAT1 phosphorylation through IFITM3. Then we examined the changes in IgE switching by applying the IFITM3 inhibitor Caraphenol A or stat1 inhibitor Fludarabine in vitro in PGRN KO mice. We found that in PGRN KO mice, the application of IFITM3 or STAT1 inhibitors restored IgE switching to WT levels, indicating that IFITM3 and STAT1 play a significant role in promoting IgE switching in PGRN KO mice, with IFITM3 and STAT1 being able to promote IgE switching (Figure 6K–N).

To further substantiate the role of PGRN in regulating IgE production through the inhibition of the IFITM3‐STAT1 signaling pathway. First, we conducted an analysis of B cell subsets in asthma patients (details of the patients are provided in Table S1, Supporting Information) and found an increase in plasma cells (Figure 6O,Q). Then we measured IgE levels in the serum of HCs and asthma patients by ELISA, finding that IgE levels were elevated in asthma patients compared to HCs (Figure 6R). Third, we extracted B cells from asthma patients and healthy controls, and after stimulating them in vitro with soluble antigens, we found that levels of pY and pBTK were elevated in asthma patients compared to HCs (Figure 6S). Additionally, we observed a decrease in PGRN expression and an increase in IFITM3 and 14‐3‐3σ expression in the B cells of asthma patients (Figure 6T). Moreover, we established a house dust mite (HDM)‐induced asthma mouse model (Figure 6P) and found that PGRN expression was also reduced in the mouse model, while IFITM3 and 14‐3‐3σ expression increased consistent with asthma patients (Figure 6T). By treating asthmatic mouse B cells in vitro with PGRN, we observed a reduction of pPI3K, pBTK, pCD19, and 14‐3‐3σ to WT levels (Figure 6U). Interestingly, the use of PGRN protein in asthma mice led to a statistically significant reduction in IgE levels in serum (Figure 6V). In summary, the data presented herein demonstrate that PGRN inhibits B‐cell activation and IgE production via the PGRN‐IFITM3‐STAT1 signaling pathway.

## 3 Discussion

In our study, we extensively explored the importance of PGRN in B‐cell signaling and its functions. Our initial findings indicated that a lack of PGRN results in notable changes in peripheral B‐cell subsets, accompanied by an increased propensity for IgE class switching. The absence of PGRN also intrinsically impacts B‐cell subsets, highlighting PGRN's intrinsic influence on B‐cell maturation. Moreover, PGRN suppression appears to enhance the early stages of B‐cell activation, mainly by influencing BCR signaling dynamics. This influence extends to wider B‐cell functionalities, with PGRN emerging as a central regulator of both BCR signaling pathways and metabolic processes. One important observation was PGRN's effect on the actin framework in B cells after initial activation, which was mediated by inhibition of the MST1‐MTOR‐STAT1‐WASP signaling pathway. The regulatory role of PGRN manifests in demonstrable effects, as evidenced by the reduced generation of NP‐specific antibodies in the absence of PGRN. Central to our findings is the exposure of the PGRN‐IFITM3‐STAT1 signaling pathway, which was pinpointed as a primary mechanism through which PGRN inhibits B‐cell activation and IgE synthesis. In summary, our data highlight PGRN's diverse and essential influences on B‐cell functions, providing key insights for the development of potential treatment approaches.

Our results align with recent transcriptomic studies that identified IFITM3 as a crucial gene involved in B‐cell activation. The upregulation of IFITM3 in PGRN KO mice further supports the idea that PGRN contributes to B‐cell activation through the regulation of key signaling genes. Notably, a recent study demonstrated that IFITM3 enhances BCR signaling by promoting BCR clustering and B‐cell activation.^[^ ^30^ ^]^ Our findings on the increased B‐cell activation and elevated pPI3K and pBTK levels in PGRN KO mice provide additional evidence for the involvement of PGRN and IFITM3 in BCR signaling modulation. Moreover, contemporary research underscores the importance of the STAT1 signaling pathway in modulating IgE synthesis in B cells.^[^ ^32^ ^]^ The observed increase in STAT1 phosphorylation in B cells lacking PGRN implies that PGRN might act as a suppressor of IgE synthesis via STAT1‐mediated mechanisms. Consequently, our results present compelling evidence suggesting a connection among PGRN, STAT1, and IgE class switching.

The interplay between IFITM3 and STAT1 exhibits complexity across different cell types. Studies have shown that in regulatory T cells (Tregs), IFITM3 can inhibit the transcription and phosphorylation of STAT1, while STAT1 can promote the transcription and expression of IFITM3^[^ ^33^ ^]^ In hepatocellular carcinoma, both IFITM3 and STAT1 are highly expressed, and there is an interaction at the protein level between the two.^[^ ^34^ ^]^ These findings suggest a potential reciprocal regulation between IFITM3 and STAT1 in different cellular contexts. In our study, we observed that in B cells of PGRN knockout (KO) mice, the application of an IFITM3 inhibitor led to a decrease in STAT1 phosphorylation, indicating a potential role of IFITM3 in inhibiting STAT1 phosphorylation. The specific details of this mechanism remain to be further investigated for clarification.

The production of IgE is tightly controlled by a delicate balance of cytokines and interactions among immune cells.^[^ ^35^ ^]^ IL‐4 promotes the differentiation of B cells into plasma cells and leads to isotype switching, a critical step in IgE production.^[^ ^36^ ^]^ In PGRN knockout mice, even though the IL‐4 secretion by iNKT (invariant Natural Killer T) cells is reduced, the overall levels of IL‐4 show no significant difference.^[^ ^37^, ^38^ ^]^ This suggests that PGRN‐mediated IgE production is a result of multifactorial interactions. Additionally, previous studies have indicated that loss‐of‐function (LOF) mutations in STAT3 lead to increased IgE levels.^[^ ^39^ ^]^ Our findings reveal that STAT3 can inhibit the expression of 14‐3‐3σ,^[^ ^20^ ^]^ a key factor in immunoglobulin class switch DNA recombination (CSR) in B cells, thus regulating IgE production.^[^ ^40^ ^]^ Signal Transducer and Activator of Transcription 1(STAT1) plays a crucial role in the immune system, especially in the regulation and response of B cells. It mediates cellular responses to interferons and other cytokines, affecting B cell activation, differentiation, and function.^[^ ^41^ ^]^ Abnormalities in STAT1 signaling, such as gain‐of‐function mutations, can lead to defects in adaptive immunity, including reduced memory B cells and altered cytokine production. These changes in STAT1 activity can influence B‐cell apoptosis, potentially leading to immunological diseases and B‐cell lymphopenia.^[^ ^42^ ^]^ Therefore, STAT1 is essential for maintaining appropriate immune responses and B cell homeostasis. Although STAT1 can mediate IgE production, its exact mechanism remains unclear.^[^ ^14^ ^]^ We report for the first time that STAT1 can directly bind to the promoter of 14‐3‐3σ, promoting its expression and thereby mediating IgE production. These findings provide further insight into the detailed mechanism by which PGRN mediates high IgE levels in bronchial asthma.

Although a marked decrease in MZ and GC B cells was observed in PGRN KO mice, there were no significant differences in the proliferation and apoptosis of these cells, as evidenced by Annexin V and Ki67 expression. This observation implies that PGRN may impact B cell differentiation through pathways beyond mere proliferation and apoptosis. The lack of PGRN may disrupt crucial signaling pathways and inflammatory regulation essential for maintaining MZ and GC B cells, resulting in their reduced numbers. These findings emphasize the critical role of PGRN in maintaining B cell homeostasis and suggest the existence of other regulatory mechanisms that require further exploration.

A limitation of our study was the utilization of a global knockout mouse model, in which the influence of other cells on B cells could not be excluded, raising questions about the role of PGRN in the broader immune system. For example, we observed that mice immunized with NP‐Ficoll and NP‐KLH produced inconsistent amounts of NP‐IgM and IgG_1_, possibly due to the combined effects of multiple immune cells, including T cells. Therefore, to further address these issues, it is necessary to further explore the effects of PGRN on the immune response of B cells using conditional knockdown of PGRN in B cells or Tfh cells. A major breakthrough made in this study is the discovery that PGRN can inhibit B‐cell activation and IgE production through the PGRN‐IFITM3‐STAT1 signaling pathway. This finding significantly expands our understanding of the regulatory roles of PGRN in the immune system and identifies potential therapeutic targets for conditions with abnormal B‐cell activation and IgE production. Despite these novel insights, studies in more extensive and diverse cohorts are necessary to validate these findings, as the complex nature of immune responses necessitates caution in generalizing these results. Moreover, additional experiments with specific inhibitors or activators could provide further mechanistic insight into the roles of the PGRN‐IFITM3‐STAT1 pathway in B‐cell activation and IgE production.

Importantly, the implications of PGRN‐mediated modulation of B‐cell function extend beyond basic immunological research. Considering the key function of B cells in antibody‐driven immune responses and the onset of autoimmune and allergic disorders, it is necessary to understand how PGRN regulates B cells, as this knowledge could pave the way for therapeutic advancements. Contemporary research indicates that PGRN concentrations vary in autoimmune diseases such as rheumatoid arthritis and systemic lupus erythematosus, potentially playing a role in their onset.^[^ ^43^ ^]^ Furthermore, a connection to PGRN has been noted in allergic conditions such as asthma and atopic dermatitis.^[^ ^19^ ^]^ Our research, emphasizing PGRN's influence on B‐cell activity and IgE synthesis, offers insights into possible focal points for the development of therapies for these medical conditions, which is particularly significant for the treatment of asthma.

In conclusion, our research underscores the vital function of PGRN in influencing B‐cell activity and IgE synthesis via the PGRN‐IFITM3‐STAT1 signaling pathway. These observations provide a holistic view of PGRN's diverse roles in B‐cell physiology and immune regulation. The regulatory strategies pinpointed in our study could pave the way for innovative therapeutic approaches for immune‐associated diseases where abnormal B‐cell responses and IgE synthesis participate in disease development.

## 4 Experimental Section

### Mice

Methods Mice C57BL/6 PGRN knockout (KO) and wild‐type were purchased from the Jackson Laboratory. The experiments were performed using the mice in a constant environment with room temperature and humidity, 12 h light cycle. Mice 6–8 weeks old were used in the experiments. All the relevant animal experiments were conducted in strict accordance with the protocols approved by the Animal Care and Use Committee of Zunyi Medical University (approval No.: ZMU21‐2202–063)

### Patients

Children explicitly diagnosed with bronchial asthma at this hospital were included in this study (*n* = 10). All children were diagnosed according to the latest national guidelines (2024 Global Initiative for Asthma (GINA) guidelines). The control group comprised age‐matched healthy children (HCs) visiting this hospital for routine health check‐ups. In this experiment, human peripheral blood mononuclear cells (PBMCs) were isolated and CD19 beads (130‐050‐301; Miltenyi) were used to select B cells. The parents of all children who participated in the research provided their written informed consent, in accordance with the Zunyi Medical University Ethics Committee (ZMU 2024‐1‐232).

### Immunizations

PBS (200 µL) containing 40 µg of NP‐KLH (N‐5060‐5; Biosearch) with an equal volume of adjuvant (Sigma) was injected intraperitoneally. On day 14, flow cytometry of the splenic lymphocytes was performed. For secondary immunization, on day 14, 40 µg of NP‐KLH/adjuvant was injected intraperitoneally, and serum samples were taken on day 28 for ELISA to measure the level of NP‐specific IgM, IgG_1_, and IgE. For the purpose of NP‐FICOLL immunization, 40 µg of NP‐AECM‐FICOLL (sc‐396292; Santa Cruz) reconstituted in 200 µl PBS with adjuvant was injected intraperitoneally, while serum samples were collected 7 days later for ELISA and flow cytometry.

### Enzyme‐Linked Immunosorbent Assay (ELISA)

Serum samples were collected from asthmatic patients and HC, PGRN‐KO mice, and WT mice, and assessed for PGRN (EK0973; BOSTER), IgE (EMC117.96; NEOBIOSCIENCE) concentrations using an ELISA kit. Serum from mice treated with NP‐KLH was collected, and NP‐specific antibodies in serum were assessed by suitably diluting the serum in wells precoated with NP‐linked bovine serum albumin (200 µg mL^−1^). This step was followed by treatment with biotin‐labeled antibodies specific for IgM, IgG, or IgE. Identification was conducted using streptavidin horseradish peroxidase combined with TMB substrate, and optical density measurements were acquired at 450 nm. All procedures, including the generation of standard curves, were strictly adhered to according to the instructions provided with each reagent.

### Asthma Model

An asthma model was established with 6 to 8‐week‐old WT mice and their corresponding control mice of a C57BL/6 background. Sensitization was done through intranasal instillation of 25 µg of house dust mite (HDM; XPB81D3A2.5; Greerlabs) extract on days 0, 1, and 2, followed by monitoring the health status of the mice. Provocation was performed on days 14, 15, 16, and 17 using 5 µg HDM extract, and mice were sacrificed 24 h after the final provocation. To investigate the role of PGRN, the mice in the asthma model were divided into three groups: PBS group, HDM group, and PGRN recombinant protein (50396‐M08H; Sino Biological) + HDM group. PGRN recombinant protein (100 ng in 20 µL PBS) was applied intranasally 1 h before HDM instillation, with a 24 h processing for collecting results.

### Flow Cytometric Analysis

PBMCs were isolated from mouse bone marrow and spleens. Nonspecific binding was blocked with an anti‐mouse CD16/CD32 antibody (BD Bioscience, San Jose, Calif). The following fluorescently labeled antibodies were employed for surface staining of cells: 7‐AAD ViabilityStaining Solution (00‐6993‐50; eBioscience), PE‐anti‐BP‐1(108307; BioLegend), PE/Cy7‐anti‐CD24(101822; BioLegend), BV510‐anti‐B220(103247; BioLegend), BV421‐anti‐IgM (406518; BioLegend), PerCP/Cy5.5–anti‐IgD (405710; BioLegend), APC–anti‐CD21(123412; BioLegend), PE–anti‐CD23(101608; BioLegend), FITC–anti‐CD95(152606; BioLegend), APC‐anti GL7(144618; BioLegend), PE‐anti CD5(100608; BioLegend), APC‐anti‐CD11b (101212; BioLegend), FITC‐anti‐CD19(115506; BioLegend), PE‐streptavidin (405203; BioLegend), PB‐anti‐CD4(100428; BioLegend), PerCP/Cy5.5‐anti‐CD44(103036; BioLegend), Biotin‐anti‐CXCR5(145510; BioLegend), PE/Cy7‐anti‐PD‐1(109110; BioLegend), APC‐anti‐CD43(143208; BioLegend), PE‐anti‐Ly‐51(108307; BioLegend), APC/Cy7‐anti‐CD45.1(110716; BioLegend), FITC‐anti‐B220(103206; BioLegend), BV510‐anti‐CD45.2(109837; BioLegend), PE–anti‐NP (N‐5060‐5; Biosearch Technologies), PerCP/Cy5.5‐anti‐B220(103234; BioLegend), PB‐anti‐IgD (405712; BioLegend), BV510‐anti‐CD138(142521; BioLegend).

For the detection of intracellular and nuclear molecules, cells were first fixed and permeabilized with the use of a FOXP3/Transcription Factor Staining Buffer Set (eBioscience, San Diego, Calif.), after surface staining, and then stained with PE–anti–IgE (406907; BioLegend), APC‐anti‐IgG_1_ (406610; BioLegend), PE/Cy7‐anti‐KI67 (25‐5698‐82; eBioscience). The experiments were carried out following the protocols provided with each reagent.

### Phos‐Flow

PBMCs from healthy controls (HCs) and asthma patients were incubated with biotin‐conjugated F(ab')_2_ Ig(M+G) (109‐006‐127; Jackson) for 30 min on ice, followed by a 10 min incubation with streptavidin. Cell activation was achieved by treating the cells for 5 and 10 min at a stable temperature of 37 °C. Subsequently, the cells were stained with APC‐anti‐CD19 (302212; BioLegend), fixed with Phosflow Lyse/Fix Buffer, and permeabilized using Phosflow Perm Buffer III from BD Biosciences. The cells were then stained with either an anti‐phosphotyrosine (pY) antibody (05321; Merck Millipore) or anti‐pBTK (5082S; CST), followed by secondary staining with Alexa Fluor® 488‐conjugated AffiniPure Donkey Anti‐Mouse IgG(H+L) (115‐475‐146; Jackson) or DyLight 405‐AffiniPure Goat Anti‐Mouse IgG(H+L) (715‐545‐150; Jackson). Data were analyzed using BD flow cytometry and FlowJo V10 software.

### Isolation and In Vitro Culture of B Cells

The spleen was harvested and carefully mashed to obtain a splenic cell suspension. B cell isolation followed a method established in previous research.^[^ ^20^ ^]^ To investigate the effect of PGRN knockout on antibody class switching, 1 × 10^6^ cells were stimulated *ex vivo* with 40 µg mL^−1^ lipopolysaccharide (297‐473‐0, Sigma), 0.2 µg µL^−1^ IL‐4 (NBP2‐35131; Novus), and 15 µg mL^−1^ CD40 (BE0016‐2‐5MG; BioXcell). The cells were cultured at 37 °C with 5% CO2 for 5 days before collection. For in vitro treatment with the STAT1 inhibitor, 5 µg mL^−1^ Fludarabine (HY‐D0715, MedChemExpress) was applied 2 h prior to culture. Internal IgE levels were evaluated via surface staining and subsequent cell fixation. Data were collected using a BD FACSVerse flow cytometer and analyzed with FlowJo software (Tree Star).

### Measurement of Calcium Flux by Flow Cytometry

Spleens were isolated as previously described,^[^ ^21^ ^]^ and purified splenic B cells (5 × 10^6^) were labeled with the calcium‐sensitive dye Fluo‐4 AM (0.5 µm; S1060, Beyotime) in Ca^2+^‐free HBSS (14175079; Gibco) for 25 min. The cells were loaded with Fluo‐4 AM and stained with a Percp‐anti‐B220(103234; BioLegend) antibody for 30 min before analysis by flow cytometry. Initial fluorescence levels were recorded for an initial 30‐second duration. Subsequently, the cells were promptly activated with preheated Biotin‐SP‐AffiniPure F(ab')_2_ Fragment Goat Anti‐Mouse IgG (10 µg mL^−1^; 115‐066‐068; Jackson) and were then analyzed using FlowJo software.

### BCR Internalization

Splenic B cells from WT or PGRN KO mice were incubated with Biotin‐SP‐AffiniPure F(ab')_2_ Fragment Goat Anti‐Mouse IgG at 4 °C for 30 min, followed by warming to 37 °C for various time intervals. The cells were then fixed, stained with PE‐streptavidin to detect any remaining surface‐bound biotinylated antibodies, and analyzed using flow cytometry.

### ChIP Assay

The ChIP assay was conducted according to the procedures reported in the literature.^[^ ^20^ ^]^ Briefly, chromatin immunoprecipitation was carried out using the SimpleChIP Enzymatic Chromatin IP Kit (Cell Signaling Technology). B cells from WT C57BL/6 mice were cross‐linked with formaldehyde for 10 min at room temperature. Sonication of cell lysates generated chromatin fragments of 100–1000 bp, which were then immunoprecipitated with an anti‐STAT1 antibody. PCR analysis was conducted using a Bio‐Rad PCR instrument with primers targeting the *14‐3‐3σ* promoter (−6179 to −6077): forward *5'*‐CACACCCACACTACCTCACA‐*3'* and reverse *5'*‐GTGGTAGTGCTGTCCAGGTG‐*3'*.

### Luciferase Reporter Assay

HEK293T cells were plated in 24‐well plates and co‐transfected with the pGL3‐14‐3‐3σ promoter‐luciferase construct along with either pAd5‐E1‐CMV or pAd5‐E1‐CMV‐STAT1. Each group also included pRL‐TK, with transfections carried out using Lipofectamine 3000. After a 48 h incubation, a passive lysis buffer was added to each well, and the supernatant was collected for analysis using Solarbio's Dual‐Luciferase Reporter Assay System.

### Immunofluorescence Analysis

Spleens from both WT and PGRN KO mice were collected for immunofluorescence analysis. Immediately after harvesting, the spleens were embedded in an OCT medium and cryosectioned to a thickness of 10 µm. The sections were fixed in cold acetone for 5 min, followed by a 15 min rinse in PBS. To block non‐specific binding, the slides were incubated with 5% bovine serum albumin and 1% anti‐CD16/CD32 monoclonal antibody (101319, BioLegend). The sections were then stained with primary antibodies, including PE‐CD4 (100407, BioLegend), FITC‐GL7 (562080, BD Biosciences), and IgD (13‐5993‐85, Thermo Fisher) in blocking buffer and incubated overnight at 4 °C. Fluorescent images were acquired using a Zeiss LSM 780 or Nikon confocal microscope.

### Immunoblotting

Immunoblot analysis was conducted following previously outlined methods.^[^ ^22^ ^]^ B cells from the spleens of WT and PGRN KO mice were isolated and stimulated on ice for 30 min with biotin‐conjugated F(ab')_2_ anti‐mouse Ig(M + G)(156736; Jackson). After incubation with streptavidin for 10 min, the cells were placed in a 37 °C water bath for 0, 5, 10, and 30 min for activation. The cells were then lysed in a strong lysis buffer containing phosphatase inhibitors A and B (G2007‐1ML; Servicebio) and a protease inhibitor (G2006‐250ul; Servicebio) on ice for 30 min. The lysate underwent centrifugation at 12000 rpm for total protein extraction. Following sodium dodecyl sulfate (SDS)‐polyacrylamide gel electrophoresis, proteins were relayed onto PVDF membranes. These membranes were then exposed to a series of antibodies, including anti‐pAKT (Ser473; CST), anti‐pBTK (5082S; CST), anti‐pCD19(3571; CST), anti‐pY (05321; Merck Millipore), anti‐pSHIP (3941S; CST), anti‐pWASP (ab5278; Abcam), anti‐pS6(4856S; CST), anti‐pFOXO1(9461S; CST), anti‐pMST1/2(49332S; CST), anti‐pMST1(3681S; CST), anti‐pIKKα/β (2697S; CST), anti‐pNF‐κB‐pP65(3033S; CST), anti‐NF‐κB‐P65(4764S; CST), anti‐pSTAT1(9167S; CST), anti‐pSTAT5(4322S; CST), and anti‐pPI3K (17366S; CST), anti‐mTOR (7C10; CST), anti‐pmTOR (5536S; CST). For measurement of total protein expression, lysed B cells from humans or mice were subjected to immunoblotting. Antibodies employed encompassed BTK (8547S; CST), SHIP(2728S; CST), WASP(sc‐13139; Santa Cruz), AKT(9272S; CST), S6(2217S; CST), FOXO1(2880S; CST), PI3K(4292; CST), CD19(3574; CST), 14‐3‐3(AF4424; Bio‐Techne), MST1(14946s; CST), DOCK8(sc292124; Santa Cruz), IKKα/β (8943S; CST), STAT1(14994; CST), STAT5(ab194898; Abcam), PGRN (AF2557; R&D), IFITM3(11714‐1‐AP; Proteintech), with β‐ACTIN serving as the loading reference. The B cells from the WT and PGRN KO mice were isolated for in vitro treatment with the IFITM3 inhibitor caraphenol A (30 µm; HY‐N3540, MCE) or DMSO. Two hours post‐treatment, the same protein extraction procedure was repeated.

### Confocal Microscopy Analysis

B lymphocytes were isolated from both WT and PGRN KO mice, followed by stimulation with soluble antigen (sAg). Prior to stimulation, the cells were pre‐treated at 4 °C with a complex consisting of AF546–monobiotinylated‐Fab'–anti‐IgG and streptavidin. After treatment, the cells were thoroughly washed and then incubated at 37 °C for the designated time periods. Following incubation, the cells were fixed, permeabilized, and stained with a range of markers, including pCD19 (ab203615; Abcam), pBTK (5082S; Cell Signaling Technology), pY (05‐321; Merck‐Millipore), pSHIP (3941S; Cell Signaling Technology), and pWASP (A300‐205A; Bethyl Laboratories). Confocal microscopy was performed, and the resulting images were analyzed using NIS‐Elements AR 3.2 software.

### TIRFM

B cells isolated from the spleens of WT and PGRN KO mice were incubated at 37 °C with membrane‐associated antigen (mAg) for varying periods. Following incubation, the cells were fixed, permeabilized, and stained with the same markers used in the confocal microscopy analysis, including pCD19, pBTK, pY, pSHIP, and pWASP. The B‐cell contact area was analyzed using Total Internal Reflection Fluorescence Microscopy (TIRFM), with interference reflection microscopy images captured and processed using NIS‐Elements AR 3.2 software. Mean fluorescence intensity (MFI) was calculated within the B‐cell contact zone, with background fluorescence subtracted for accuracy. Data were collected from over 30 cells across 2 to 3 independent experiments.

### Bone Marrow Chimaeric Mice

Bone marrow samples were collected from CD45.2 WT/PGRN KO mice and CD45.1 WT mice. A 1:1 mixture was prepared by combining bone marrow cells from either CD45.2 WT or CD45.2 PGRN KO mice with those from CD45.1 WT mice. This cell mixture was then intravenously injected into CD45.1 recipient mice that had been preconditioned with a sublethal radiation dose of 6 Gy to establish bone marrow chimeras. After 8 weeks of bone marrow reconstitution, splenic cells were harvested for further analysis.

### BrdU Incorporation and Staining

Mice were administered intraperitoneal injections of BrdU at a dose of 10 mg^−1^ kg^−1^ day^−1^ for 24 h prior to sacrifice. Splenocytes were subsequently isolated and stained with antibodies targeting various cell surface markers. BrdU incorporation was assessed using the BrdU Flow Kit (BD, 557892) according to the manufacturer's protocol.

### Quantitative RT‐PCR

Total RNA was extracted from splenic B cells of WT and PGRN KO mice using Trizol Reagent (BioTeke). The extracted RNA was then reverse‐transcribed into cDNA using the PrimeScript RT Reagent Kit (Takara, US), following the manufacturer's guidelines. Quantitative gene expression analysis was performed using specific primer pairs on a CFX96 Real‐Time PCR System (Bio‐Rad). *Ifitm3* promoter forward: *5ʹ*‐TAGCCTATGCCTACTCCGTGAAGTC‐*3ʹ*, reverse: *5ʹ*‐CTGAGGACCAAGGTGCTGATGTTC‐*3ʹ*.

### Seahorse Assay

Twenty‐four well microplates were initially coated with 50 µg mL^−1^ poly‐D‐lysine (C0132; Beyotime) and incubated overnight at 4 °C. Splenic B cells (2 × 10^6^) were then exposed to 10 µg mL^−1^ LPS or 10 µg mL^−1^ F(ab′)_2_ Ig(M+G) for 2 h before being transferred to Seahorse 24‐well plates. The cells underwent three washes with Seahorse Assay Medium, which contained 25 mm glucose (G8769; Sigma), 2 mm L‐glutamine (G6392; Sigma), and 1 mm sodium pyruvate. Following this preparation, the oxygen consumption rate (OCR) was measured under standard conditions using the Seahorse XF24 Extracellular Flux Analyzer, particularly after the sequential addition of 1.5 µm oligomycin (abs42024304; Absin) and 1 µm antimycin A.

### B‐Cell Proliferation Assay

B cells were isolated and labeled with CellTrace Violet (CTV; C34557; Thermo Fisher) at a concentration of 5 × 10^5^ cells mL^−1^. Following this, they were stimulated with 10 µg mL^−1^ Biotin‐SP‐AffiniPure F(ab')2 Fragment Goat Anti‐Mouse IgG (109—006–127; Jackson), 10 µg mL^−1^ anti‐mouse CD40 (BE0016‐2, BioXcell), 5 µg mL^−1^ LPS (L2880; Sigma), or 10 µg mL^−1^ CpG (tlrl‐1826‐1; InvivoGen). The subsequent data analysis was conducted using a Thermo Fisher Attune NxT flow cytometer, and the results were interpreted with FlowJo V10 software from Tree Star.

### RNA Extraction and RNA‐Seq Analysis

Purified B lymphocytes were isolated from the splenic lymphocytes of WT and PGRN KO mice by using a Mouse B Lymphocyte Enrichment Set‐DM Kit (557792; BD), adhering to the manufacturer's instructions. RNA was extracted from the cells with TRIzol reagent (10296028; Invitrogen), and sequencing was then conducted with the BGIseq‐500 system. Poly(A) mRNA, enriched using oligo(dT)‐conjugated magnetic beads, was fragmented and reverse transcribed using random N6 primers to synthesize double‐stranded DNA. Subsequent smoothing and phosphorylation of the DNA ends facilitated the amplification of ligation products via PCR utilizing specific primers. Subsequent thermal denaturation of the PCR products allowed the amplification of single‐stranded DNA using a bridging primer to generate a single‐stranded circular DNA library. The reads obtained from WT and PGRN KO B cells were cleaned utilizing SOAPnuke software and were then aligned using HISAT and Bowtie2. Gene expression levels were computed using the RNA‐seq with expectation–maximization method and the differentially expressed genes were subjected to biological pathway classification and enrichment analyses, with a *Q* value of ≤ 0.05 post‐FDR correction deemed to indicate significant enrichment.

### Statistical Analysis

An unpaired *t*‐test was employed to compare two groups, while analysis of variance (ANOVA) was used for evaluating multiple groups. Statistical significance was determined using GraphPad Prism 8, with data presented as means ± SEM. A P value of less than 0.05 was considered statistically significant. Notations: *p < 0.05, **p < 0.01, ***p < 0.001, ****p < 0.0001.

## Conflict of Interest

The authors declare that they have no conflict of interest.

## Author Contributions

P.Z., C.R., and G.Y. have contributed equally to this work. P.P.Z. drafted the manuscript. P.P.Z. was responsible for data analysis and figure generation. Z.X.H., Z.C.D., and C.H.L. designed the research and performed manuscript review and revision. C.S.R. carried out the ELISA, Confocal, TRIFm, and immunofluorescence experiments. P.P.Z. was responsible for RNA extraction, RT‐PCR, and RNA‐Seq analysis. C.S.R. was responsible for Seahorse experiments. P.P.Z., Y.N.G, Y.Z, Q.L, Y.A., and G.Y.L. performed the flow cytometry assay and western blotting. P.P.Z. and G.L.Y. carried out the calcium flux, B cell proliferation assay, and BrdU incorporation and staining. P.P.Z. and Y.N.G. were responsible for the chip assay, X.D. was responsible for the Luciferase Reporter Assay, and P.H. and Y.C. assisted with the manuscript.

## Supporting information



Supporting Information

Supporting Information

## Data Availability

The data analyzed in the current study is available from the corresponding author on reasonable request.
