# Supplementary material for: PGRN Inhibits Early B‐cell Activation and IgE Production Through the IFITM3‐STAT1 Signaling Pathway in Asthma
Source: Adv Sci (Weinh). 2024 Oct 16;11(45):2403939. doi: 10.1002/advs.202403939 (PMC11615816; doi:10.1002/advs.202403939)
Supplement: Supplementary file 1 — Supporting Information [file ADVS-11-2403939-s002.pdf]

ADVANCED SCIENCE
Open Access

Supporting Information

for *Adv. Sci.*, DOI 10.1002/advs.202403939

PGRN Inhibits Early B-cell Activation and IgE Production Through the IFITM3-STAT1 Signaling Pathway in Asthma

Pingping Zhang, Changshun Ruan, Guangli Yang, Yaning Guan, Yin Zhu, Qian Li, Xin Dai, Yang An, Xiaoqi Shi, Pei Huang, Yan Chen, Zhixu He, Zuochen Du* and Chaohong Liu**

Figure S1

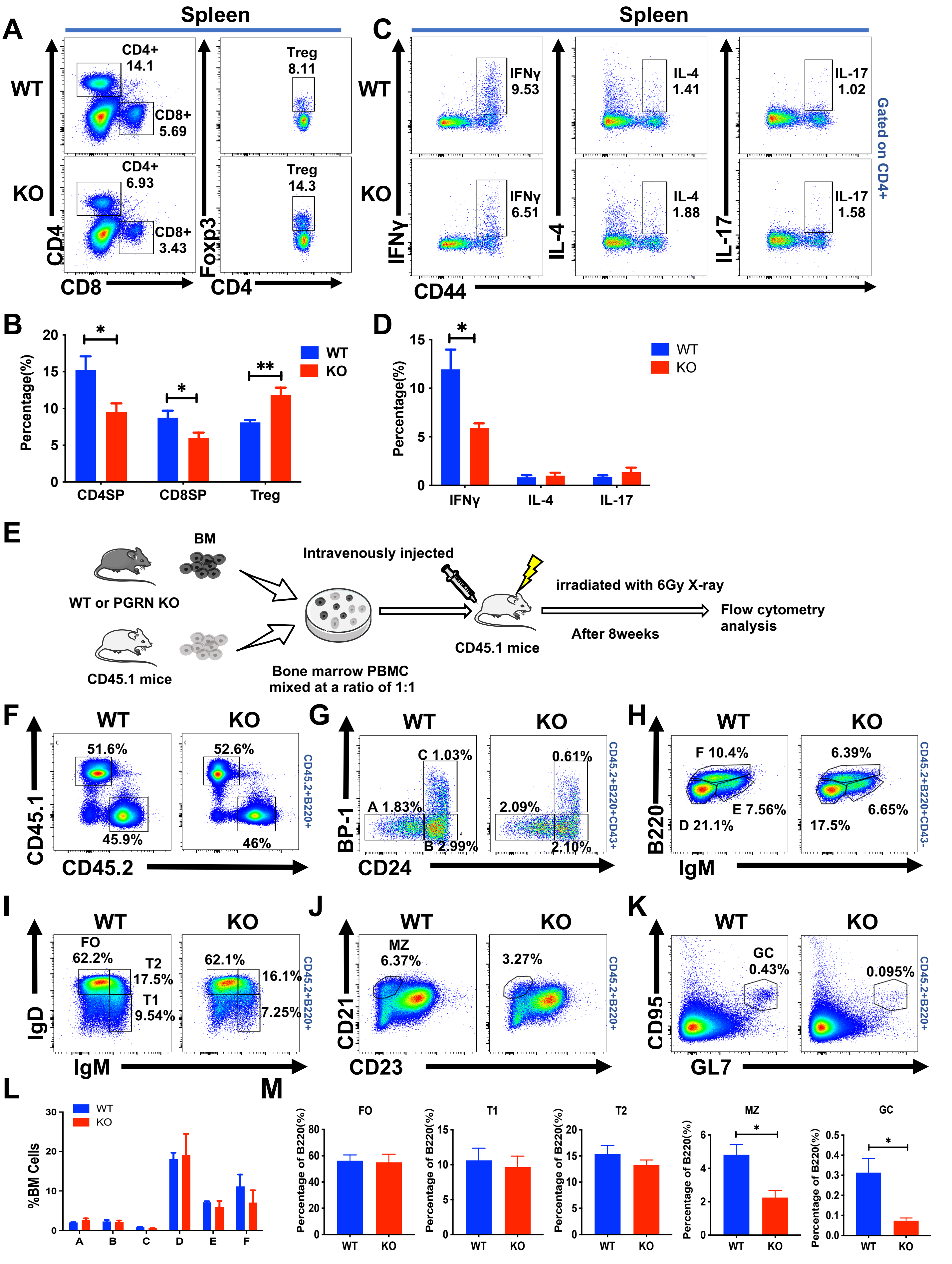
A
Spleen
WT
KO
CD4+
14.1
CD8+
5.69
CD4+
6.93
CD8+
3.43
Treg
8.11
Treg
14.3
CD4
CD8
Foxp3
CD4
C
Spleen
IFNγ
9.53
IFNγ
6.51
IL-4
1.41
IL-4
1.88
IL-17
1.02
IL-17
1.58
Gated on CD4+
IFNγ
IL-4
IL-17
CD44
B
Percentage(%)
WT
KO
CD4SP
CD8SP
Treg
D
Percentage(%)
IFNγ
IL-4
IL-17
E
BM
WT or PGRN KO
CD45.1 mice
Intravenously injected
Bone marrow PBMC
mixed at a ratio of 1:1
CD45.1 mice
irradiated with 6Gy X-ray
After 8weeks
Flow cytometry
analysis
F
WT
KO
51.6%
45.9%
52.6%
46%
CD45.1
CD45.2
CD45.2+B220+
G
C 1.03%
A 1.83%
B 2.99%
0.61%
2.09%
2.10%
BP-1
CD24
CD45.2+B220+CD43+
H
F 10.4%
E 7.56%
D 21.1%
6.39%
6.65%
17.5%
B220
IgM
CD45.2+B220+CD43-
I
FO
62.2%
T2
17.5%
T1
9.54%
62.1%
16.1%
7.25%
IgD
IgM
J
MZ
6.37%
3.27%
CD21
CD23
K
GC
0.43%
0.095%
CD95
GL7
L
%BM Cells
A
B
C
D
E
F
M
FO
T1
T2
MZ
GC
Percentage of B220(%)

Figure S2

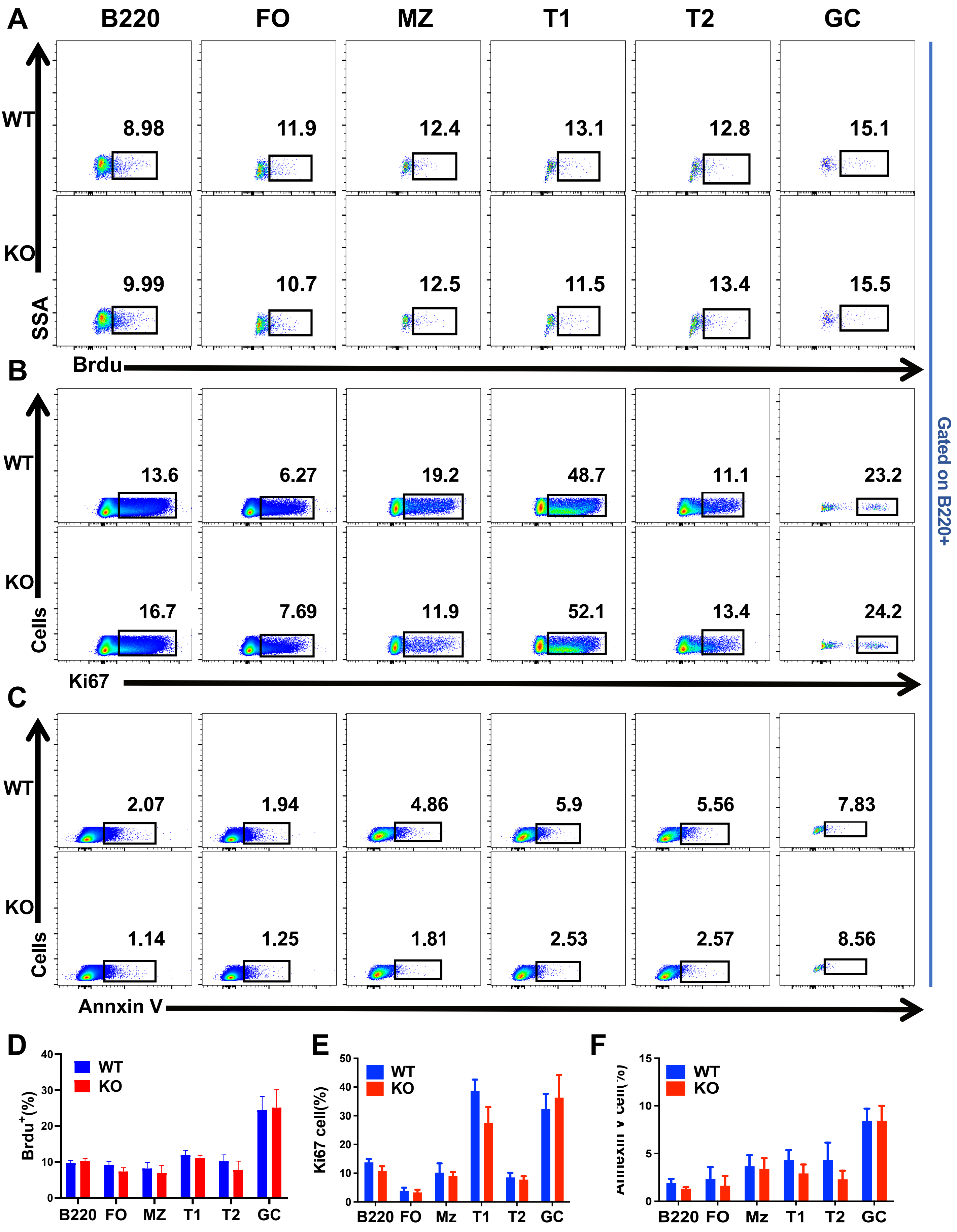
A
B220
FO
MZ
T1
T2
GC
WT
KO
SSA
8.98
11.9
12.4
13.1
12.8
15.1
9.99
10.7
12.5
11.5
13.4
15.5
Brdu
B
WT
KO
Cells
13.6
6.27
19.2
48.7
11.1
23.2
16.7
7.69
11.9
52.1
13.4
24.2
Ki67
C
WT
KO
Cells
2.07
1.94
4.86
5.9
5.56
7.83
1.14
1.25
1.81
2.53
2.57
8.56
Annxin V
Gated on B220+
D
WT
KO
Brdu+(%)
0
10
20
30
40
B220
FO
MZ
T1
T2
GC
E
WT
KO
Ki67 cell(%)
0
10
20
30
40
50
B220
FO
Mz
T1
T2
GC
F
WT
KO
0
5
10
15
B220
FO
Mz
T1
T2
GC

Figure S3

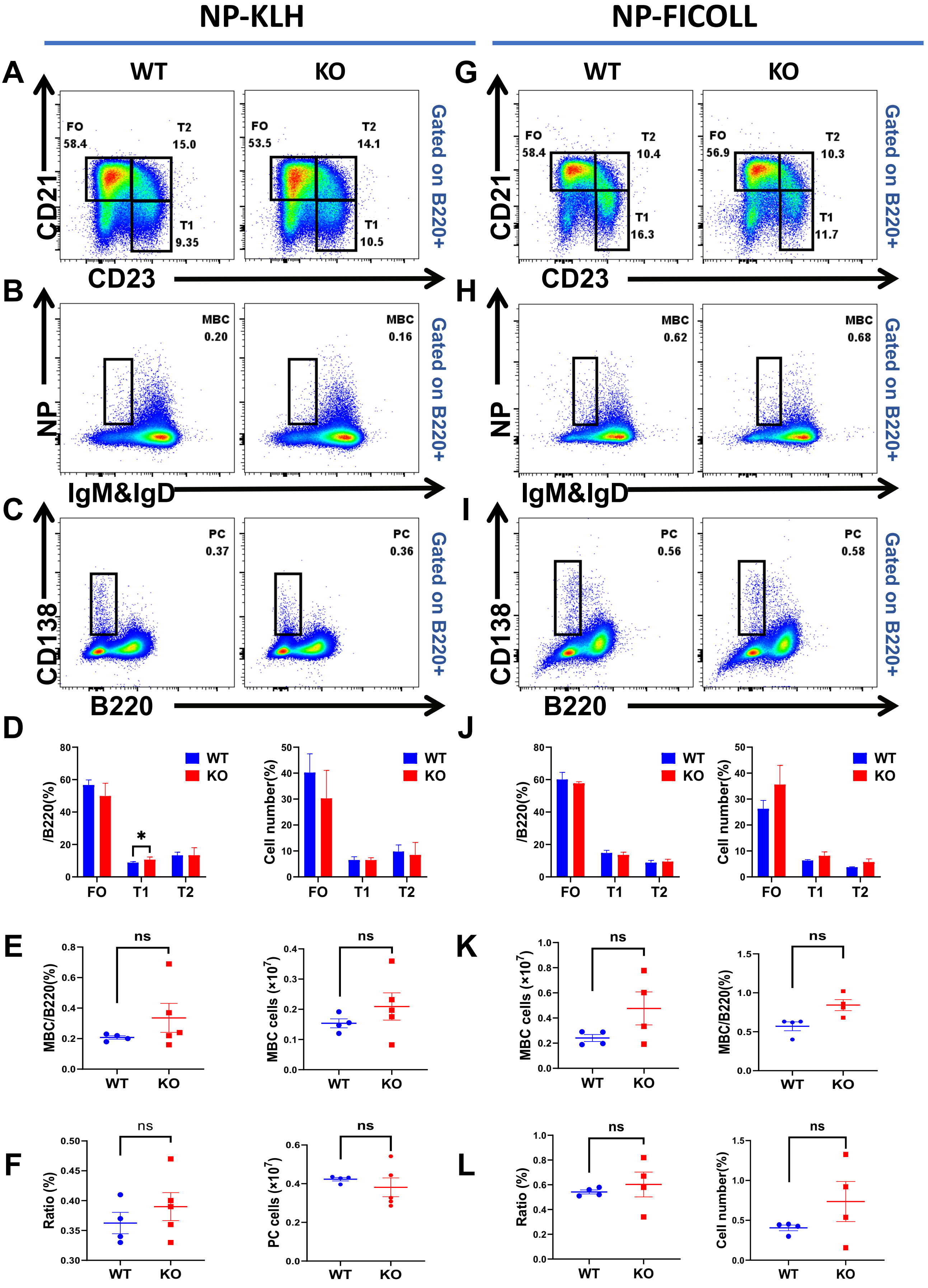
NP-KLH
NP-FICOLL
A
G
WT
KO
FO 58.4
T2 15.0
T1 9.35
FO 53.5
T2 14.1
T1 10.5
FO 58.4
T2 10.4
T1 16.3
FO 56.9
T2 10.3
T1 11.7
CD21
CD23
Gated on B220+
B
H
MBC 0.20
MBC 0.16
MBC 0.62
MBC 0.68
NP
IgM&IgD
C
I
PC 0.37
PC 0.36
PC 0.56
PC 0.58
CD138
B220
D
J
/B220(%)
Cell number(%)
FO
T1
T2
WT
KO
*
E
K
ns
MBC/B220(%)
MBC cells (×10^7)
F
L
Ratio (%)
PC cells (×10^7)
Cell number(%)

Figure S4

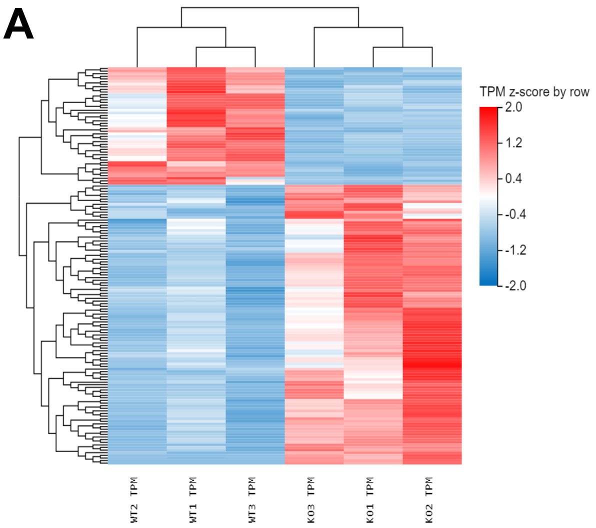
A
TPM z-score by row
2.0
1.2
0.4
-0.4
-1.2
-2.0
WT2_TPM
WT1_TPM
WT3_TPM
KO3_TPM
KO1_TPM
KO2_TPM

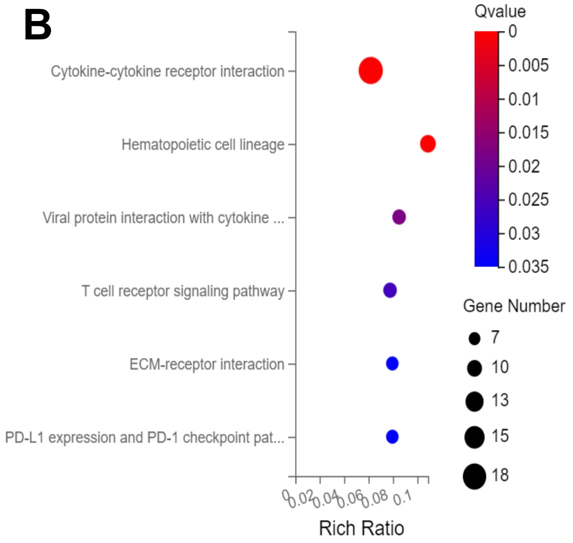
B
Qvalue
0
0.005
0.01
0.015
0.02
0.025
0.03
0.035
Cytokine-cytokine receptor interaction
Hematopoietic cell lineage
Viral protein interaction with cytokine ...
T cell receptor signaling pathway
ECM-receptor interaction
PD-L1 expression and PD-1 checkpoint pat...
Gene Number
7
10
13
15
18
0
0.02
0.04
0.06
0.08
0.1
Rich Ratio
